# The ESTHER hospital partnership initiative: a powerful levy for building capacities to combat the HIV pandemic in low-resource countries

**DOI:** 10.1186/s12992-016-0149-9

**Published:** 2016-04-01

**Authors:** Gilles Raguin, on behalf of the French ESTHER network

**Affiliations:** Expertise France, 73 rue de Vaugirard, Paris, 75006 France

**Keywords:** Hospital partnership, Capacity building, HIV/AIDS, Low income countries

## Abstract

Partnerships between hospitals in high income countries and low resource countries are uniquely capable of fulfilling the tripartite needs of care, training, and research required to address health care crises in low resource countries. Of particular interest, at a time when the EBOLA crisis highlights the weaknesses of health systems in resource-poor settings, the institutional resources and expertise of hospitals can also contribute to strengthening health systems with long-term sustainability.

We describe a partnership network between French Hospitals and hospitals/health structures in 19 countries that demonstrates the power and efficacy of health partnership in the response to the HIV/AIDS pandemic in sub-Saharan Africa and south East Asia. Through the ESTHER initiative, the partnership network currently provides capacity development, care and treatment to over 165,000 HIV-positive patients at 87 urban and 92 peripheral sites in 17 countries and enrolls 19,000 new HIV positive patients, delivers psychosocial services to 120 000 people and tests more than 35,000 pregnant women for HIV annually. It also, engages communities and assists with the development of a robust electronic information system.

Launched in 2002, the ESTHER (Ensemble pour une Solidarite Thérapeutique Hospitalière En Reseau) initiative has grown from small projects with a focus on access to antiretroviral treatment in a limited number of West African countries at its outset into a large and comprehensive HIV/AIDS-control system in Western and Central Africa. The partnership’s rapid achievements in the fight against HIV/AIDS, combined with the comprehensive and long-term approach to countries’ health care needs, suggest that this “twinning” and medical mentoring model can and should be duplicated and developed to address the ever more pressing demand for response to global health needs in low resource countries.

## Article

Gaps and inequities in health persist both within and between countries, underscoring a failure to share advances in health equitably [1]. The World Health Report 2006 particularly emphasized the global health workforce crisis, calling for action on Human Resources for Health (HRH) (WHO [2]). Improving service coverage and health outcomes is conditional on an adequate, competent, equitably distributed, well supported health workforce. Whilst increasing the number of health workers is part of the solution, improving the capacity of the existing workforce through continuous education and professional development, mentoring, peer support, professional and knowledge networks, training and post-graduate education is essential.

Few low income countries have comprehensive systems of continuing education so clinical mentoring has been adopted as one approach to strengthen clinical skills and yield sustainable quality clinical outcomes. The World health Organization (WHO) has been recommending clinical mentoring in antiretroviral treatment (ART) roll-out since 2005 [2], combined with standardized, simplified clinical protocols and standard operating procedures.

The focus of the ESTHER (Ensemble pour une Solidarite Thérapeutique Hospitalière En Reseau) initiative on strengthening the capacity of health professionals through peer to peer partnerships places it within the global effort to address the HRH crisis. ESTHER partnerships are contributing to continuing professional development (CPD), postgraduate education, multidisciplinary training, mentoring and peer support.

But ESTHER partnerships also provide an avenue for health providers from wealthier countries to demonstrate their solidarity with and provide assistance to their colleagues and their patients in poor resource countries. This solidarity became an ethical duty in the early 2000s when, finally, after lengthy discussions among the international community, access to antiretroviral therapy became possible for millions of patients dying of AIDS in the developing world.

With the advent of the Global Fund and the President’s Emergency Plan for AIDS Relief, the availability of antiretrovirals (ARVs) increased drastically - 10 million people living with HIV/AIDS (PLWHA) in 2012 - and ESTHER partnerships developed in 19 countries, simultaneously providing services, manpower mobilization, clinical mentoring, teaching, and conducting operational research. Over the last 10 years, hundreds of French healthcare providers have responded meaningfully to the acute health needs of Sub-Saharan Africa in the face of the HIV/AIDS crisis - one of the most devastating pandemics in human history - while coping with weak health systems and a dramatic lack of qualified staff.

In this article, we describe the ESTHER initiative, a replicable model of hospital partnership between French hospitals and their African and Asian counterparts, able to implement a successful and comprehensive system to build and develop capacities for controlling the HIV/AIDS crisis in low-resource countries. We describe the characteristics of the partnership, the responses to the challenges faced in building and sustaining the system, the lessons learned and the power of this model.

### Working through partnership and the capacity development approach

Emerging approaches for addressing knowledge and capacity gaps are moving away from the traditional avenues commonly focused on donor-driven short term gap-filling. They place greater emphasis on empowerment and leadership by the beneficiary country, the use of country systems, and peer to peer exchanges of experiences, with southern countries increasingly involved as skill providers [3].

The ESTHER initiative provides such a partnership approach to capacity development between northern and southern practitioners and institutions and through promoting south-south learning. ESTHER offers a large range of capacity building techniques, from conventional methods such as workshops and seminars, exchange visits, training of trainers, post-graduate medical education programs and short term technical assistance to more recent methods such as mentoring, developing professional networks, e-learning and telemedicine.

The WHO working definition of partnership is “a collaborative relationship between two or more parties based on trust, equality and mutual understanding for the achievement of a specified goal” [4]. At its best, partnership through development cooperation addresses some of the shortcomings of traditional technical assistance; it is a locally owned, long term commitment, creating enabling environments for innovation and learning. Shared accountability is critical as well as monitoring and evaluating the partnership’s quality. Attaining this quality is a complex process that requires time to develop and can be unappealing for donors who usually prefer time-bound projects.

#### The ESTHER model of hospital Partnership

At its inception in 2002, the main hospitals in Senegal, Benin, Burkina Faso, Cambodia, Mali, Vietnam, and Cameroon - the seven countries in which the ESTHER initiative began - had very few doctors trained in HIV care; they were seeking expatriate clinical teachers and institutional partnerships to help tackle the epidemic. Meanwhile infectious diseases specialists from different French university hospitals with volunteer experience in developing countries had started teaching African colleagues and sending limited numbers of ARVs.

Prompted by these developments and calls for action from civil society, the French minister of health, Dr Bernard Kouchner, set up the ESTHER initiative and provided the ministry’s help in mobilizing the expertise and solidarity of French care givers in the direction of Africa. The main focus of the initiative at the time was on access to antiretroviral treatment. Capacity building and clinical mentoring formed the framework for this HIV-control program.

**Fig. 1:**
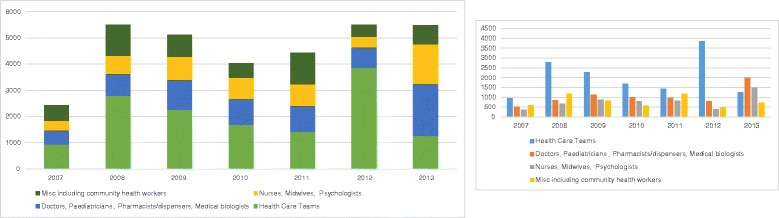
Profile of Health staff trained, 2007-2013

Counterpart relationships at both individual and departmental levels were the keystone of the partnerships. All disciplines involved in HIV/AIDS care were involved, from doctors to nurses, laboratory technicians, psychologists, epidemiologists and community health workers. Hospital managers and cost controllers were also drawn in to increase the management capacities of partner hospitals (Fig. 1).

After initial success with the first partnerships, ESTHER sought to have a wider impact on HIV care, mainly in western and central Africa. Since 2002, 48 French hospitals institutions have participated in the ESTHER initiative in 19 countries. The initiative currently includes 33 University hospitals and 12 General Hospitals in all regions of France. In total, over the last 10 years, more than 34 000 Africans and Asians caregivers have participated in exchange with and mentorship of hospital staff, trainees and students. The partnerships have had a major impact on the delivery of health services and operational research in West and Central Africa (WCA), including more than 30 jointly authored publications [5–11].

One of the fundamental tenets of the ESTHER partnership is that all participants expect and work for mutual benefit. African hospitals benefit from the expertise, capacities and financial commitment from the ESTHER network of French hospitals while French staff benefits personally and professionally from involvement in the program.

There is substantial evidence that this large and sustained initiative is more useful for public health than frequent, short-term focused initiatives that usually fail to facilitate sustainable improvements in the developing country’s health care system and tends to develop artificial white elephants.

The long-term ESTHER capacity-building effort also stands in contrast to short-term commitments from individual health care workers traveling to developing countries from the western world. These short term efforts can offer value to both the health care providers and the patients they serve but they do not contribute significantly to the building of developing countries’ health care systems.

Such initiatives should be largely promoted and developed since they are uniquely capable of fulfilling the tripartite needs of care, training, and research required to foster the health of individuals and their communities in low resource countries. Disappointingly, however, funding is difficult to elicit for such initiatives and we advocate strongly for directing more support and resources to long-term hospital partnerships that contribute to health system building.

#### The ESTHER Model for Care and Treatment of HIV/AIDS

The tragic scope of the HIV/AIDS pandemic is well known. In 2013, an estimated 35 million people worldwide were living with HIV, and an estimated 1.5 million lost their lives to AIDS.

The once-high cost of antiretroviral drugs, along with concerns about therapy adherence and the possible negative effect of antiretroviral therapy on risk behaviors, posed barriers to widespread HIV/AIDS treatment in sub-Saharan Africa. Many of those concerns have been addressed in recent years, and delivery of antiretroviral therapy has been successful in many settings in Africa [12, 13].

However, sustaining effective antiretroviral therapy and controlling HIV/AIDS in sub-Saharan Africa remains a substantial challenge due to issues such as poverty, hunger, gender discrimination, stigma and the poor quality of health services. Establishing and maintaining a proper system of care is especially difficult in these countries, which claim 60 % of the world’s HIV/AIDS burden but can call on only 1.3 % of the world’s health care workforce to tackle it [14].

**Fig. 2:**
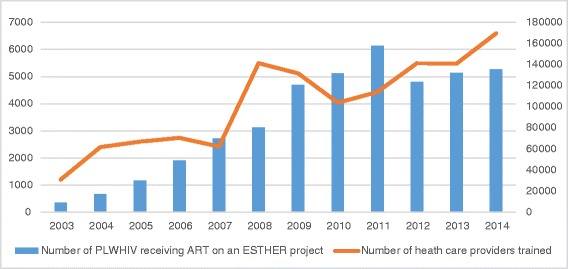
Patient recruitment and professional training, 2003-2014

In this context, ESTHER has progressively developed a comprehensive HIV/AIDS-capacity building model, providing a complete system of care that has proved to be adaptable and sustainable in all countries. Delivery of services occurs in the public sector through hospitals and health centers run by Ministries of Health. Through a network of community-based organizations linked to the public health system, ESTHER extends its impact to wide geographic areas, sometimes covering all regions of a country such as Cameroon. In this particular example, ESTHER has, over the last 10 years, contributed to the treatment of over 49,000 HIV-positive patients at 13 urban and 22 outlying clinical sites, currently enrolling nearly 5,000 new patients in the country annually (Fig. 2) [15]. Quality of care monitoring has recently become a central issue, with the use and development of viral load testing and adherence support programs in order to improve HIV control and retention of patients into care [16].

#### Continuum of care and community support

In all the health settings in which the initiative began, right from the outset we systematically underpinned our hospital partnerships with a component of community support. Set up with the help of existing community-based organizations or through the development of new ones, the presence of persons living with HIV/AIDS in their governance and membership bodies was a pre-requisite. We knew from previous experience that supporting public or private health structures was not enough for scaling up testing, access to ART and, above all, retention. We also knew that the stigma associated with HIV/AIDS and poor economic and literacy status were major obstacles to access to care; patient and continued work in and with the community were thus a condition to success. Community mobilization, health and treatment education, adherence support and training and development of counselors and peer educators appeared to be strong predictors of success for testing, care and treatment, as well as changes to HIV/AIDS practices and policies in hospitals.

Occasionally, in some of our programs, partner community organizations were supported to help particularly poor patients (70 % of HIV patients lived with less than 1 $ per day in the early years of the initiative) with food donations, the development of income generating activities and support for orphans and vulnerable children born to HIV-positive parents.

Community support was also critical to developing specific, tailored interventions for hard to reach key populations, such as men having sex with men (MSM), sex workers, prisoners and drug users. In a number of countries, the ESTHER model has proven highly efficient in improving access to care and treatment for these most at risk groups.

Hence, over the 10 years of the initiative, more than 130 community-based organizations (CBOs) were supported financially and technically so as to acquire the capacity to provide services and become eligible for international or local funding.

#### Decentralization of HIV care and treatment

Given the rising numbers of patients being treated (outstripping the capacities of the treatment sites receiving support) and the context of global advocacy for scaling-up access to ARV, we decided - along with the national authorities - to scale up access to care and treatment in many of our partner countries via the decentralization of capacities to additional facilities in more regions and districts. In some countries, such as Benin or Cameroon, ESTHER was able to provide national coverage by multiplying hospital partnerships and contributing to the development of a national framework of training of trainers. ESTHER has also contributed to the development of task shifting in some countries, a solution that has had difficulties emerging in WCA, contrary to Eastern and southern Africa where it is now well accepted. This strategy led us, in a number of poorly resourced countries, to develop south-south partnerships.

#### HIV/AIDS Information systems

The use of information and communications technologies (ICT) for improving health services in low income countries has recently been emphasized as a critical point [17]. The lifelong nature of HIV/AIDS care, monitoring patient adherence to antiretroviral therapy, the need for reliable quantification of medication and tests needs and operational research demands accurate and detailed record keeping, a significant barrier to adequate funding and sustainable care in the developing world.

On these grounds, and because no electronic records system was available in WCA in response, ESTHER decided to create such a system. We did so in partnership with Epiconcept, an organizational off-shoot of Medecins sans frontières (MSF) that provides technical assistance to medical aid organizations working in low resource countries. This system, called ESOPE, has evolved into a shareware electronic medical record system that can be used for adults, HIV-positive mothers and their children and can be networked in a country in order to provide critically-needed aggregated national information data. To date, ESOPE is being used by five countries as the national reference system for HIV monitoring and by 3 additional countries on specific treatment sites [18].

#### Operational research (OR)

Operational and implementation research play an important role in fostering innovation and developing better models for the implementation of effective programs. A partnership model, with involvement of local program staff, is more likely to generate successful implementation of research findings [19]. The ESTHER initiative has been well positioned to develop and promote operational research and effective implementation models, with university medical staff involved in most ESTHER interventions. Through its own network of university hospitals or through partnerships with research institutions such as the French National Agency on Aids Research (ANRS), the Institute for Research and Development (IRD) and the Pasteur Institute, partners have been able to produce 138 conference abstracts and more than 20 publications in medical reviews over the last ten years, contributing to improved global health through evidence-based effective interventions.

#### Transparency, accountability and funding

**Fig. 3:**
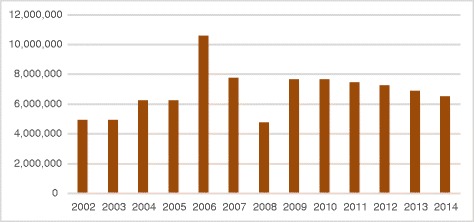
Cost of production (Euros)

Building up medical capacities has just been one part of the story. One of the challenges we also faced was developing the administrative capacity required to properly manage national staff and grants received for the initiative - from the French government or other sources. Assuring fiscal accountability and measuring financial performance in order to match donors’ criteria has been particularly challenging at a time where transparence and accountability have become a priority. We managed by opening offices in all countries in which our programs have expanded and/or benefited from significant volumes of funding (Fig. 3).

## Discussion

Although combating the HIV epidemic calls for complex interfaces between different types of actors and interventions, hospital partnerships have proven highly successful in supporting and developing the capacities of numerous health actors. A large number of health providers and health institutions included in the partnership network have become HIV/AIDS care and national training reference centers. Hospital partnerships have made significant contributions to accelerated scale-up of ART in most countries, and were the first sites to make ART available in many of them. Today, the ESTHER initiative receives grants from UNITAID, the French Development Agency, the French government, the Global Fund for AIDS, Tuberculosis and Malaria through different national bodies, the city council of Paris. French public hospitals provide their invaluable in-kind support and demonstrations of solidarity.

### Strengthening health systems

It is worthy of note that beyond the HIV/AIDS response, the partnership network has had collateral impact on the broader strengthening of health systems. Indeed, in the real world of low resource countries, HIV/AIDS infection is not an isolated disease with its specific health structures and staff. Health providers do not work uniquely with AIDS patients. They deliver care for all types of diseases. Furthermore, due to the long term consequences of HIV/AIDS, clinicians have to tackle cardiovascular, renal and metabolic complications as well as the emergence of cancers, all of which pose problems that spread far beyond HIV patients alone. Building laboratory and health information unit capacities, strengthening the management and supply of HIV commodities, improving standards of care, patient safety, governance and accountability can easily extend and, in reality does just that, to all aspects of health. Through the ESTHER initiative, hospitals and health systems have begun to access a broad array of expertise and funding sources, providing support and development to the system in its entirety. This support, along with support for programmatic and faculty development in multiple disciplines, plays a role in increasing national capacity to address wider health needs while improving the working conditions of health providers – acutely needed to stem the dramatic brain drain.

Finally, it is important to stress the role of ministries of health in initiating and sustaining the partnerships. At inception, our hospital partnerships often resulted from personal, departmental and institutional commitments. However, all ESTHER programs, when validated, start with the signature of a Memorandum of Understanding between the French ministry of health and the national ministry of health. The programs are integrated into national programs and action plans; they also aim to help meet ambitious public health goals. As it has transpired, they play a highly useful catalytic role in triggering cross-institution cooperation and social mobilization.

### The Power of Hospital Partnerships

Although debate is still raging on best practice approaches to strengthening health care systems in the developing world [20], the ESTHER experience provides a model for institutional partnerships meeting the challenge of providing health care in resource-poor environments. The success of the ESTHER initiative lies in its ability to facilitate a rapid increase in services and resources to meet the treatment needs of thousands of HIV patients at multiple clinical sites in a large number of WCA countries. With time, ESTHER has also been able to help decentralize care in rural and regional settings and provide a comprehensive system of care in an environment hosting training and research. These results are directly attributable to the substantial resources created by the medical and CBO partnerships.

The current HRH crisis facing sub-Saharan Africa demands a response from the international medical community in developed countries. African hospitals need support with service delivery, teaching, and operational research. For our part, we must engage in a sustainable and equitable relationship and help create leverage to secure the most needed resources from national budgets and international funders.

The ESTHER initiative began in response to the HIV/AIDS epidemic, and its response has been rapid, comprehensive and effective. It has evolved in a remarkable and flexible way, staying fully in step with health systems’ changing needs. We call on WHO and donors who support the challenges of global health and its post-2015 agenda to recognize and support the potential of hospital partnerships for a meaningful response.

### Limitations

Despite its potential and successes, the initiative does have limitations. The engagement of local and national health authorities is critical for ensuring health workers have the resources needed to deliver proper care. The initiative itself does not supply them (medicines, reagents, equipment).

The enduring motivation and stability of local health workers involved in the initiative is also critical for the durability of results. Yet working conditions are largely unappealing in many low resource countries and the unstemmed flight of quality personnel represents a serious, albeit not crippling, limit to this type of program.

Lastly, analyzing and evaluating the results of such an initiative is often difficult, as is the case for so many vertical or cross-sector international aid and development programs. This makes it hard to credit results to one specific intervention.

## Conclusion

The ESTHER initiative is a successful and dynamic initiative implemented on behalf of the French government. ESTHER’s support for HIV prevention, care and treatment has had major positive effects on the health of beneficiaries, on institutions and systems in partner countries and on the overall global response to HIV.

The ESTHER initiative is also addressing the HRH crisis which is recognized by donors as a vital determinant of global health inequities. Its partnership and capacity development approaches are at the heart of current thinking in best practice.

ESTHER also contributes to health systems, strengthening and improving health outcomes through its partnership programs.

The post millennium development goals (MDG) health landscape provides opportunities for ESTHER to continue and expand its capacity development to new themes and new implementation models that enhance systems and the capacity of partner countries to sustainably manage the response to HIV and other health crisis.

Finally, ESTHER has recently joined a new agency of the French government, EXPERTISE FRANCE, which will continue to support its activities and development.
